# Pollen Processing Behavior of *Heliconius* Butterflies: A Derived Grooming Behavior

**DOI:** 10.1673/031.011.9901

**Published:** 2011-08-09

**Authors:** Anna-Laetitia Hikl, Harald W. Krenn

**Affiliations:** University of Vienna, Department of Evolutionary Biology, Althanstrasse 14, A-1090, Vienna, Austria

**Keywords:** evolution, feeding mechanism, Lepidoptera, proboscis movements, video analysis

## Abstract

Pollen feeding behaviors *Heliconius* and *Laparus* (Lepidoptera: Nymphalidae) represent a key innovation that has shaped other life history traits of these neotropical butterflies. Although all flower visiting Lepidoptera regularly come in contact with pollen, only *Heliconius* and *Laparus* butterflies actively collect pollen with the proboscis and subsequently take up nutrients from the pollen grains. This study focused on the behavior of pollen processing and compared the movement patterns with proboscis grooming behavior in various nymphalid butterflies using video analysis. The proboscis movements of pollen processing behavior consisted of a lengthy series of repeated coiling and uncoiling movements in a loosely coiled proboscis position combined with up and down movements and the release of saliva. The proboscis-grooming behavior was triggered by contamination of the proboscis in both pollen feeding and non-pollen feeding nymphalid butterflies. Proboscis grooming movements included interrupted series of coiling and uncoiling movements, characteristic sideways movements, proboscis lifting, and occasionally full extension of the proboscis. Discharge of saliva was more pronounced in pollen feeding species than in non-pollen feeding butterfly species. We conclude that the pollen processing behavior of *Heliconius* and *Laparus* is a modified proboscis grooming behavior that originally served to clean the proboscis after contamination with particles.

## Introduction

Nectar consuming butterflies come into contact with pollen while visiting flowers. When butterflies search for and consume nectar on flowers, pollen frequently adheres to the proboscis, head, or other body parts. However, only butterflies of the closely related neotropical genera *Heliconius* and *Laparus* (Lepidoptera: Nymphalidae) evolved a feeding technique in which amino acids are extracted from the pollen grains (Gilbert 1972; Boggs et al. 1981; Estrada and Jiggins 2002; O'Brien et al. 2003). Pollen as an additional source of nutrition is central in their life histories, and is linked to other elaborated life history traits such as extended longevity, mutualistic insect-plant interactions, uninterrupted ovogenesis, and cyanogenesis in the adults and larvae (Gilbert 1972, 1991; Boggs et al. 1981; Engler et al. 2000; Beltran et al. 2007).

Due to the high nutritional quality of pollen grains that contain amino acids, proteins, polysaccharides, lipids, and sometimes vitamins (Baker 1978; Baker and Baker 1986) there can be no doubt about the advantages of using pollen as a food source. Most flower visiting arthropods consume pollen by chewing and mastication, or they swallow whole pollen grains (e.g. Simpson 1955; Smith and Mommsen 1984; Crailsheim et al. 1992; Van Rijn and Tanigoshi 1999; Krenn et al. 2005, 2008; Momose 2005; Karolyi et al. 2009). However, butterflies, which possess a suctorial proboscis that serves to ingest fluids, need a special technique to utilize the nutritional content of pollen. The behavior is termed “pollen feeding”, and the technique of nutrient acquisition is referred to as “pollen processing behavior” (Gilbert 1972) because the pollen is not ingested, but undergoes a special treatment on the outside of the proboscis by which the butterfly extracts nutrients from the pollen grains (Gilbert 1972; O'Brien et al. 2003; Krenn et al. 2009).

**Figure 1.**
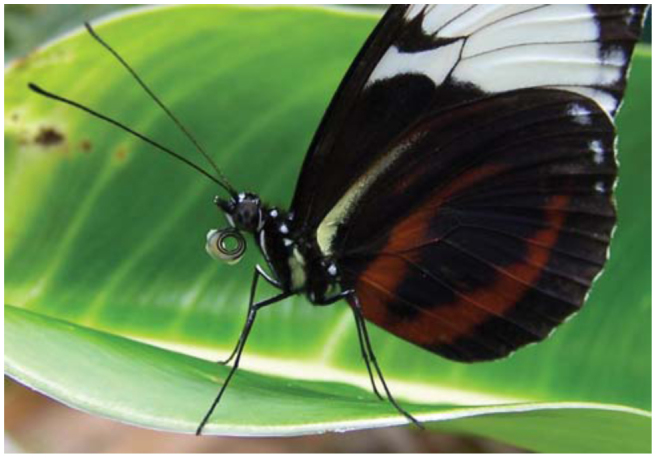
*Heliconius cydno* sitting motionless and processing pollen with coiling and uncoiling proboscis movements. The coiled position of the proboscis was coded as extension category I; (greenhouse population in Brackenridge Field Laboratory of the University of Texas; photo: courtesy of S. H. Eberhard.). High quality figures are available online.

Butterflies of the genera *Heliconius* and *Laparus* actively collect pollen during flower probing and accumulate it on the outside of the basal third of the proboscis (Boggs et al. 1981; Penz and Krenn 2000), where specialized bristle-shaped sensilla retain the pollen load (Figure 1) (Krenn and Penz 1998). The pollen load is subsequently processed by coiling and uncoiling movements of the proboscis that can last several hours (Gilbert 1972, 1975; Boggs 1987). During this behavior, these butterflies release a fluid from the proboscis that is repeatedly imbibed and re-released. Amino acids are extracted from the pollen grains and taken up via the ingested fluid (O'Brien et al. 2003). During pollen processing, the pollen grains become damaged (Krenn et al. 2009) and their contents are released into the extracting fluid, which has been shown to be saliva (Eberhard et al. 2009a). After completion of pollen processing, the pollen falls off the proboscis (Gilbert 1972). Given that proteolytic enzymes in the saliva (Eberhard et al. 2007) are responsible for the extraction, the processing of pollen on the proboscis constitutes an unusual example of extra-oral digestion.

Although nitrogenous components of the pollen grains have long been regarded as the basis for the evolution of novel life history traits in *Heliconius* butterflies (Gilbert 1991), the evolutionary origins of the pollen processing behavior remain obscure. In this study, the proboscis movements of the pollen processing behavior in species of *Heliconius* were described using video analysis and movements were compared to those of related nymphalid butterflies which were exposed to small pollen sized particles. In nymphalid butterflies, contamination of the body (antennae, proboscis, and eyes) with small particles is known to release grooming behavior performed by the mid-tibia (Jander 1966). However, observations of neotropical butterflies in the field (Nilic and Lintner, unpublished) indicated that proboscis movements also eliminate small particles from the proboscis, and that saliva was used to clean the food canal of the proboscis. Based on these observations, we hypothesized that pollen processing behavior is a derived proboscis grooming behavior that allows *Heliconius* butterflies to utilize the pollen adhering to the proboscis as a source of nutrient.

## Materials and Methods

### Analysis of pollen processing behavior in *Heliconius* butterflies

Videos of pollen processing behavior were recorded in *Heliconius* butterflies from a greenhouse population in Brackenridge Field Laboratory of the University of Texas (Austin, USA) in April 2008. A total of 3 hours and 40 min were recorded in 5 individuals of *Heliconius cydno* (Doubleday 1847) and 6 individuals of *Heliconius melpomene* (Linnaeus) (Lepidoptera: Nymphalidae). Prior to filming, these butterflies voluntarily collected pollen from flowers of *Psiguria (=Anguria)* sp. (Curcurbitales: Cucurbitaceae), a plant that is known to be a primary source of pollen for *Heliconius* butterflies (Gilbert 1972; Boggs et al. 1981; Murawski and Gilbert 1986; Estada and Jiggins 2002; Krenn et al. 2009).

### Proboscis grooming behavior

The comparative study of the proboscis movements was carried out in the Tropical Research Station La Gamba in Costa Rica (8° 45' N, 83° 10' W; 81 m asl) between February to April 2007. The ecosystem diversity and the butterfly fauna of this region were described in Weissenhofer et al. (2008). The butterflies were caught with a net in the surrounding habitats of the station next to the Bosque Esquinas (Piedras Blancas National Park).

Proboscis movements were recorded in four pollen feeding species: *Heliconius hecale* (Fabricius), *Heliconius sara* (Fabricius), *Heliconius pachinus* (Salvin), and *Laparus doris* (Linnaeus), as well as in three non— pollen feeding nymphalid butterflies: *Eueides lybia* (Fabricius), *Dryas iulia* (Fabricius), and *Anartia fatima* Fabricius. From each species, five to six individuals were tested; a total of 39 individuals were video recorded. In all butterflies, proboscis movements were triggered by contamination of the proboscis with small particles. Glass beads (diameter 106 μm and finer, Sigma, www.sigmaaldrich.com) were placed on the butterfly's proboscis using an insect pin in two subsequent trials. Each individual was subjected to the following sequence: (1) the butterfly was fed with water in an insectarium ten minutes before each trial; (2) glass beads were placed on the proboscis and the reaction was recorded for 20 min; (3) the proboscis was rinsed and the butterfly was set free. In some cases, when a butterfly constantly moved around or showed no reaction to the contamination material, the observation was stopped.

### Behavioral analysis

**Figure 2.**
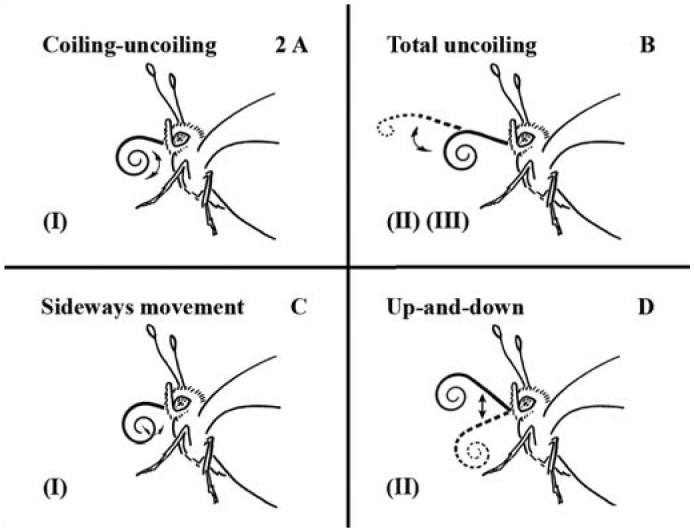
Proboscis movements in the loosely coiled and uncoiled positions; extension categories I–III. (A) The coiling and uncoiling movement pattern; arrows show directions of movements of the proboscis. In category I, the uncoiled proximal part of the proboscis is shorter than the diameter of the proboscis spiral. (B) The uncoiling movement; arrows show the uncoiling of the proboscis to its totally extended position (dotted line). The loosely coiled proboscis shows extension category II and the uncoiled proboscis shows extension category III. (C) The sideways movement. Arrows indicate lateral movements of the coiled proboscis. (D) The up and down movement pattern; arrows show the upward and downward motions of the proboscis; it is raised and lowered at the joint to the head. High quality figures are available online.

A hard disc camcorder (JVC GZ-MG37E, www.jvc.com) was used to record 20 min after the first mouthpart movements in each individual (N = 50). All videos were saved on an external hard disc in avi AVI and MPEG formats. All movements of each butterfly were analyzed using “The Observer XT” software (© 2005 Noldus Information Technology) (Noldus 1991). All butterflies were filmed in lateral or semi-lateral views to ensure a detailed behavioral analysis. Distinct patterns of proboscis movements were distinguished, i.e., coiling, uncoiling and sideways movement, degrees of proboscis extensions, as well as release of fluid (Figure. 2).

The proboscis extensions were coded into three categories. In category (I), the proboscis was tightly coiled; the proximal part of the proboscis was extended less than the diameter of the proboscis spiral in a totally coiled position (Fig. 2A) (Video). In category II, the diameter of the loose proboscis spiral and exceeded the diameter in coiled position by more than a quarter (Figure 2B). In category III, the proboscis was more or less straightly uncoiled (Figure 2D).

**Figure 3.**
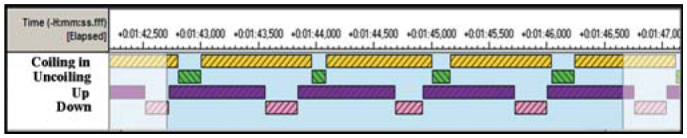
Fine scale analysis of pollen processing behaviour in *Heliconius cydno*. Screenshot showing an example of 4 sec from Observer XT. Four proboscis movements are represented, i.e. coiling, uncoiling, up and down. The coiling and uncoiling motions occured simultaneously with the up and down movements of the proboscis. All movements were repeated four times in the same order. High quality figures are available online.

A fine scale analysis was made from 2 min recordings of the pollen processing behavior in *H. cydno* (N = 5) and *H. melpomene* (N = 6). These analyses included only the third to the fifth minute in all 20 min recordings of pollen-processing behavior to obtain a standard set of proboscis movements that characterize natural pollen processing behavior (Figure 3). In this detailed behavioral analysis, the movements of the proboscis were broken down and coded into individual movements (i.e. coiling, uncoiling, up, down) (Figure 3).

Periods of no movement (i.e. pauses) were coded as well. A pause was defined when the entire proboscis did not move for at least one second. To avoid losing data on the duration of each behavior, all movements were coded as state events in Observer XT with a code for the start and the end of the behavior. The primer dataset was exported, and statistics were calculated using SPSS software, Version 11.5 of the SAS System for Windows 2002. Since the butterflies moved freely during the video recordings, some individuals turned to a position from which the proboscis movements could not be observed. These time periods made up less than 15% (except two individuals with approximately 45%) of the total observation time, and were included as “movements not observable” in the data analyses.

Non-parametric statistics were used to compare the data. Significance of the comparisons was set at *p* < 0.05 level.

## Results

### Pollen processing behavior

During pollen processing behavior, individuals of *H. cydno* and *H. melpomene* sat motionless with their wings closed upwards. They moved the proboscis to handle pollen from *Psiguria* sp. flowers that adhered to the coils during previous foraging bouts (Figure 1). Pollen processing behavior was a repeated sequence of partly coiling and uncoiling movements of the proboscis (Figure 2A). At the same time, saliva was released and mixed with the collected pollen load. A repetitive sequence of the several proboscis movements, i.e. coiling, uncoiling, up and down (Figure 3) was regularly detected in pollen processing behavior of *H. cydno* and *H. melpomene*. Depending on the size of the pollen load, the proboscis was coiled from a fully coiled to a loosely coiled position (equivalent to extension category I and II). During the coiling movement, the number of coils increases, and the proboscis spiral became tighter. During uncoiling the number of coils decreases, and the diameter of the proboscis spiral widens. At the same time, the entire proboscis is raised upwards at its joint to the head and is lowered afterwards, resulting in an up and down movement sequence.

**Video 1.**
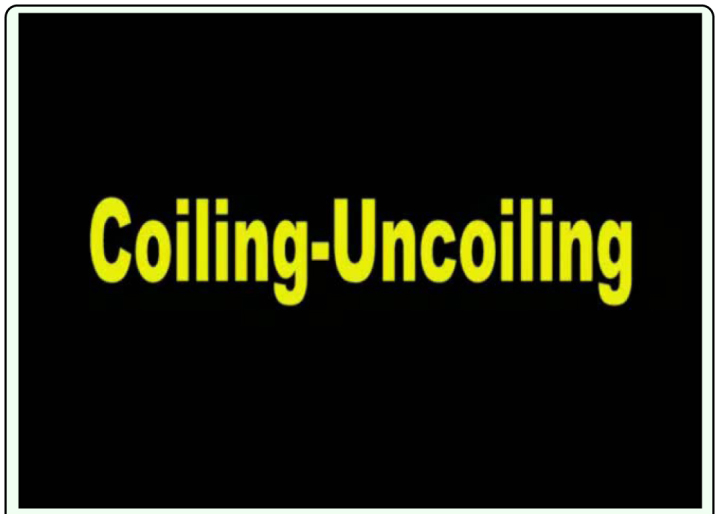
Movements of the proboscis during pollen proccessing in *Heliconius cydno*. Click image to view video. Download Video.

**Figure 4.**
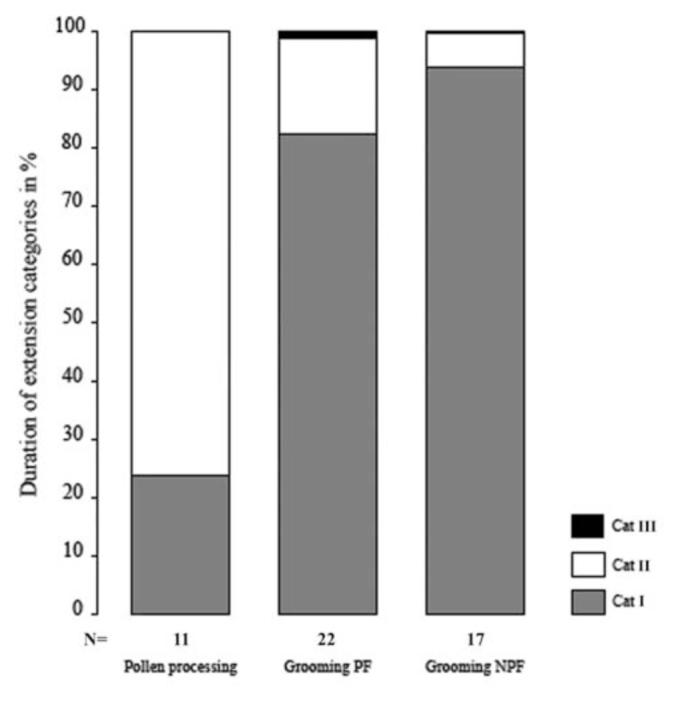
Position of the proboscis during pollen processing in pollen feeding *Heliconius* butterflies (N = II) and during proboscis grooming behavior in pollen feeding (PF) Heliconiini (N = 22) and non-pollen feeding (NPF) Nymphalidae (N = 17). Total duration in percent of observed proboscis extension categories I, II, and III per 20 min video recordings. High quality figures are available online.

Due to the size of the pollen load, some specimens were unable to fully coil the proboscis into resting position. They processed the pollen in proboscis extension category II. Proboscis extension category III was never observed during pollen processing behavior (Figure 4).

The characteristic cycle of proboscis movements during pollen-processing are displayed in Figure 3 as an example of the fine scale analysis. An upward movement of the proboscis was accompanied by simultaneous and quick uncoiling, after which a lengthier period of coiling followed. Coiling behavior occupied the majority of total time (60.5 %) per 2 min (N = 11). A rapid downward movement of the proboscis initiated proboscis coiling, after which the whole pattern would begin anew with the upward uncoiling movements (Fig. 3). During the uncoiling motions of the proboscis, the inner coils were in contact with the outer coils and they slid over the pollen load spreading the saliva released from the proboscis.

The proboscis movement cycles in the fine scale analysis showed a mean frequency of 44.4±21.1 times per minute for coiling and uncoiling and 45.1±25.9 times per minute for up-and-down movements (Figure 3). In the up-and-down movement pattern, the proboscis spiral was elevated at the joint to the head up to 45° over the horizontal plane, after which it was subsequently lowered.

**Figure 5.**
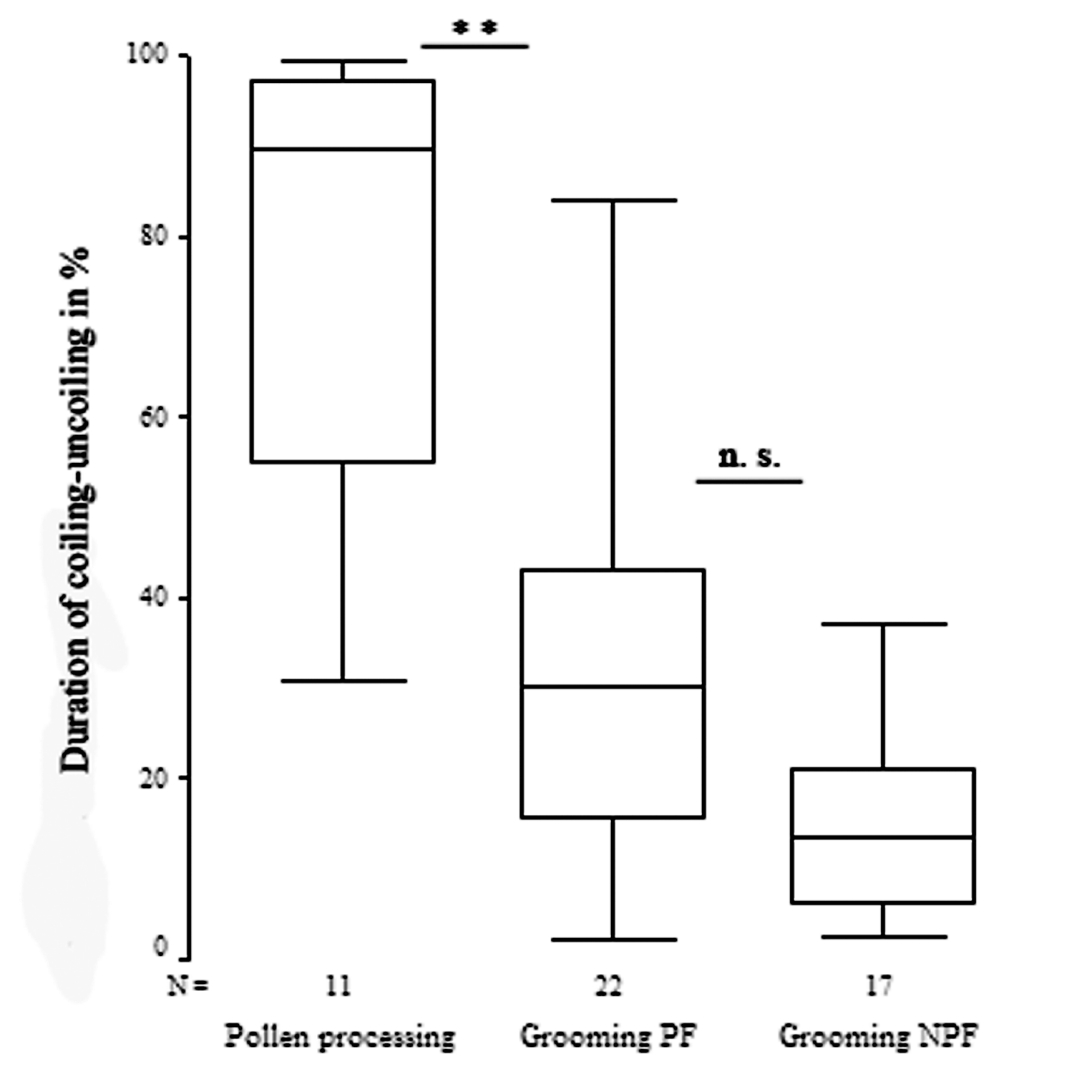
Duration of the coiling-uncoiling movements in percent of 20 min video. Coiling and uncoiling movements lasted longer in pollen processing than in grooming behavior (Mann-Whitney U-test, Z = -3.591, P < 0.0001), no significant difference of duration during grooming behavior between *Heliconius* (grooming PF) and non-pollen feeding Nymphalidae (grooming NPF) (Mann-Whitney U-test, Z = 1.785, P = 0.222). ** = highly significant; n. s. = not significant. High quality figures are available online.

In addition, sideways movement of the proboscis was found 13 times in a total of 220 min with a mean total duration of 0.54% per 20 min of video recordings of the pollen processing behavior (Figure 5). During the sideways movement, the degree of coiling remained constant, but the proboscis spiral turned to the left and right sides alternatively.

The release of saliva was observed during pollen processing behavior. Salivary fluid was released from the proximal part and the tip of the proboscis to form a thick suspension of pollen. The liquid fraction was sucked in, and again, saliva was released and added to the pollen load.

Some movement pauses occurred in most individuals between the movement cycles, described above. Pauses were observed with a mean duration of 4.18 sec, which represented about 16.06% of the 2 min fine scale analysis.

### Proboscis movements after contamination with glass beads

The proboscis grooming behavior included coiling and uncoiling, up and down, and sideways movement (Video). These movements were performed in various proboscis extension categories (Figure 2). The proboscis sometimes was uncoiled into fully extended position after contamination with glass beads (Figure 4).

In addition, the coiled proboscis was regularly moved to the lateral sides. This sideways movement consisted of a series of repeated turning motions to the left and right sides. Maximal duration of these lateral swinging movements continued up to 70 sec in *A. fatima*. Sideways movements were never performed when the proboscis was uncoiled (extension category III).

During grooming behavior, saliva discharge led to a moist proboscis surface in most of the observed specimens. Although all observed butterfly species released a liquid from the proboscis, only the *Heliconius* species released enough to form well-defined drops that could be counted. In *Heliconius* species, 1–3 drops of saliva were present at the same time on the proboscis. *Laparus doris* produced the highest number of drops of all observed species (mean 20.2 drops/20 min with glass beads) after proboscis contamination. The highest mean duration of drops was observed in *H. pachinus* after glass bead contamination with 54.1 sec per 20 min video recordings.

**Table 1.**
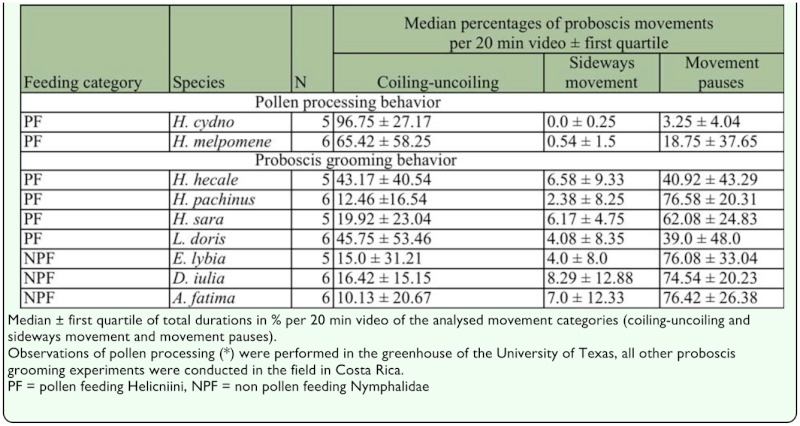
Proboscis movements of pollen processing (*) and proboscis grooming behavior.

### Pollen processing behavior versus proboscis grooming behavior

**Figure 6.**
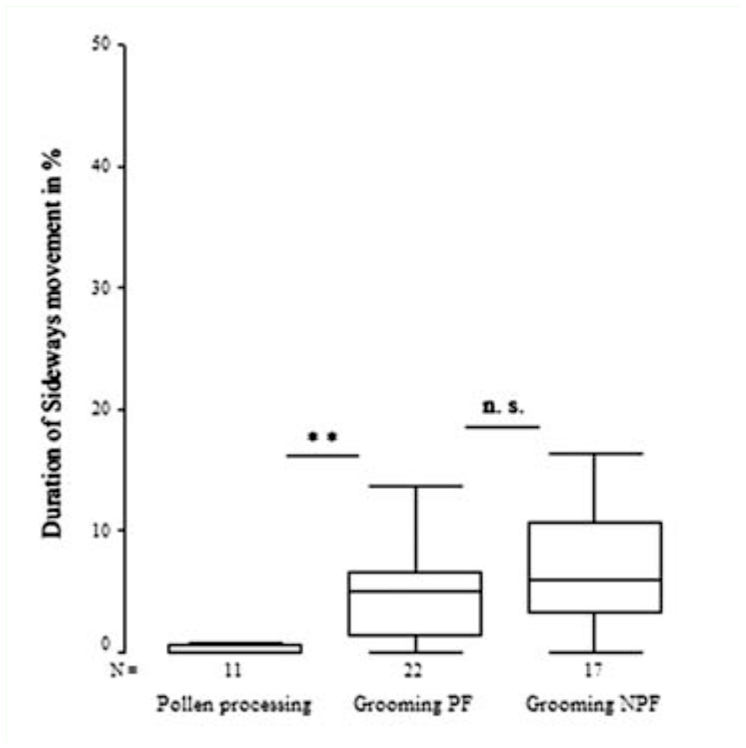
Duration of sideways movements in percent of 20 min video. Sideways movements were performed longer in proboscis grooming than in pollen processing (Mann-Whitney U-test, Z = 3.218, P = 0.003). There was no difference between pollen feeders (PF) and non-pollen feeders (NPF) in grooming behavior (Mann-Whitney U-test, Z = -0.71, P = 1.434). ** = highly significant; n. s. = not significant. High quality figures are available online.

All proboscis movements of pollen processing and proboscis grooming were compared in 20 min video recordings (Figures 4–6) (Table 1) and checked for the presence of fluid. All butterflies exhibited the same principle pattern of proboscis movements during pollen processing and proboscis grooming behaviors, including the distinct pauses in which the proboscis remained motionless and the discharge of salvia.

Regardless of feeding category (PF, NPF), the differences between pollen-processing and proboscis grooming behaviors mainly concerned (1) degree of proboscis extension (Figure 4), (2) movement pauses, and (3) sideways movements (Table 1).

The proboscis sometimes was uncoiled into the fully extended position only during proboscis grooming behavior (Figure 4), but never in pollen processing behavior.

During pollen processing, *Heliconius* butterflies performed nearly uninterrupted coiling-and-uncoiling movements together with a few pauses (Figure. 5). Pauses in movement characterized the grooming behavior of butterflies from both feeding categories (PF, NPF). The duration of movement pauses was significantly higher in grooming behavior of butterflies with an experimentally contaminated proboscis compared to pollen processing behavior of *Heliconius* butterflies (Table. 1) (Kruskal-Wallis, χ^2^ = 24.081, df = 2, *p* < 0.0001). The pauses in proboscis movements occurred mostly when the proboscis was in the coiled position (extension category I). No pauses of movement were observed in the uncoiled proboscis of extension category III.

*Heliconius* butterflies showed by far the shortest total duration of sideways movements in pollen-processing behavior (Figure 6). A long duration of sideways movement was found in all individuals tested with glass beads, and was characteristic of grooming behavior. The total duration of sideways movement was higher during proboscis grooming behavior in both groups (pollen-feeding and non-pollen feeding butterflies) compared to pollen-processing behavior of *Heliconius* (Kruskal-Wallis, χ^2^ = 13.240, df =2, *p* < 0.001).

## Discussion

Pollen-feeding in *Heliconius* butterflies is regarded as a key evolutionary feature in their life-histories (Gilbert 1972, 1991; Brown 1981; Boggs et al. 1981; Beltran et al. 2007). These insects gather pollen from flowers, but do not ingest it. Instead, the butterflies extract amino acids from pollen by destroying the grains during the pollen-processing behavior which is performed by the proboscis (Krenn et al. 2009). In this study, the stereotypic pattern of proboscis movements that occur during pollen-processing were analyzed. Pollen-processing was characterized by uninterrupted, lengthy coiling-and-uncoiling movements and up-and-down motions of the coiled proboscis. Contamination of a butterfly's proboscis with glass beads triggered proboscis grooming behavior, which is similar in many aspects to pollen processing behavior. Coiling-and-uncoiling movements occurred but pauses of movement, lateral sideways motions and uncoiling to the fully extended position of the proboscis, were regularly observed during proboscis grooming.

All butterflies released some amount of fluid during proboscis grooming and pollenprocessing behaviors. However, drops of saliva were only observed on the proboscis in *Heliconius* and *Laparus* butterflies during grooming behavior. Apparently, when the pollen load on the proboscis was very large, drops of the fluid were not visible. Instead, the fluid immediately mixed with the collected pollen during pollen-processing.

The presence of fluid on the proboscis tip was observed during pollen collecting on flowers (Penz and Krenn 2000), as well as during pollen and glass bead processing in the first investigation of pollen feeding by *Heliconius* butterflies (Gilbert 1972). A recent study proved that this fluid is saliva (Eberhard et al. 2009a). It has been established that the salivary glands of *Heliconius* are larger than in other nectar feeding butterflies and in related genera (Eberhard et al. 2009b). The present results indicate that saliva is also used for proboscis grooming, which could be the evolutionary origin of pollen processing behavior. In *Heliconius* butterflies, a special salivary pump was detected that serves for the two-way flow of fluid in the food canal of the proboscis (Eberhard and Krenn 2003). Release of saliva is not restricted to Heliconiinae and was also found in fruit feeding butterflies, which dilute dried up fruit juice before taking it up (Knopp and Krenn 2003). Likewise, the uptake of pyrrholizidin alkaloids from withering plants in Ithomiinae and Danainae (Boppré 1981, 1983) presumably involves the release of saliva from the proboscis tip.

The proboscis movements can be explained by the functional mechanism of the lepidopteran proboscis (reviewed in Krenn 2010). According to this model, the elasticity of the cuticle coils the proboscis into a loosely coiled spiral. Further coiling movements are caused by contractions of the intrinsic galeal muscles within the lumen of the proboscis. In Heliconiinae, these intrinsic galeal muscles were found to be particularly numerous and they extend further into the tip region than in other Nymphalidae (Krenn and Mühlberger 2002; Bauder and Krenn 2009). The coiling and uncoiling movement involve two different mechanisms, the intrinsic galeal muscles causes coiling and the elasticity uncoils the proboscis into a loose spiral. A hydraulic mechanism is responsible for further uncoiling, which results from increased haemolymph pressure generated by compressing the stipes pumps in the butterfly's head (Schmitt 1938; Bänziger 1971; Krenn 1990; Wannenmacher and Wasserthal 2003). The sideways movements of the proboscis are probably caused by alternative contractions of intrinsic galeal muscles in the two proboscis halves. Similar lateral movements have been observed during the proboscis assembly after emergence from the pupae (Krenn 1997). Sideward movements characterize the last phase of proboscis assembly in which the galeae shift against each other until the linking structures of the proboscis halves are locked. The observed up and down movements are probably due to contraction extrinsic galeal muscles in the basal proboscis joint and an antagonistic stipital muscle (Eastham and Eassa 1955; Krenn 1990, 2000). The latter proboscis movements are regularly seen during probing movements of the proboscis in all Lepidoptera (Krenn 1990; Penz and Krenn 2000; Krenn 2008). Thus, in principle, all individual movements of pollen processing or proboscis grooming behavior can be performed by all butterflies.

We conclude that pollen processing behavior of *Heliconius* and *Laparus* originated from grooming behavior, similar to that found in other nymphalids, but was modified by (1) the loss of the sideway bending of the coiled proboscis, (2) loss of full uncoiling, and (3) increasing periods of coiling and uncoiling motions. A similar hypothesis has been proposed for the evolution of pollen collecting behavior in bees. In these insects, leg movements are primarily responsible for pollen manipulation (i.e. pollen uptake, loading and unloading). They have been shown to represent evolutionarily derived grooming movements, which are similar to cleaning behavior (Jander 1976; Michener et al. 1978).

The evolutionary origin of pollen feeding in butterflies has involved behavioral modifications like proboscis grooming (shown in this study) and flower visiting (Penz & Krenn 2000; Krenn 2008), as well as morphological adaptations of the proboscis (Krenn & Penz 1998) and the salivary glands (Eberhard & Krenn 2003; Eberhard et al. 2009b). Which of these modifications arose first in the evolution of pollen feeding is still unknown. However, the hypothesis of the evolutionary origin of pollen processing behavior from proboscis grooming could be a valuable key toward understanding the puzzling evolution of pollen feeding. Utilization of pollen has allowed *Heliconius* butterflies and the closely related species, *Laparus doris*, to enter a novel adaptive zone in the evolution of their life histories.

## Abbreviations

NPF,: non-pollen feeders;
PF,: pollen feeders
